# Dietary Black Seed Effects on Growth Performance, Proximate Composition, Antioxidant and Histo-Biochemical Parameters of a Culturable Fish, Rohu (*Labeo rohita*)

**DOI:** 10.3390/ani11010048

**Published:** 2020-12-29

**Authors:** Maria Latif, Mehwish Faheem, Asmatullah, Seyed Hossein Hoseinifar, Hien Van Doan

**Affiliations:** 1 Department of Zoology, University of the Punjab, Lahore 54590, Pakistan; asmat_ullah.zool@pu.edu.pk; 2 Department of Zoology, Government College University, Lahore 54000, Pakistan; mehwishfaheem@gcu.edu.pk; 3 Department of Fisheries, Faculty of Fisheries and Environmental Sciences, Gorgan University of Agricultural Sciences and Natural Resources, Gorgan, Iran; Hoseinifar@gau.ac.ir; 4 Department of Animal and Aquatic Sciences, Faculty of Agriculture, Chiang Mai University, Chiang Mai 50200, Thailand; 5 Science and Technology Research Institute, Chiang Mai University, 239 Huay Keaw Rd., Suthep, Muang, Chiang Mai 50200, Thailand

**Keywords:** antioxidant, black seed, growth, oxidative stress, thymoquinone, medicinal plants

## Abstract

**Simple Summary:**

Stress-related losses are of major concern in aquaculture practices. Black seed is a medicinal plant species widely used as natural antioxidants and hepatic-nephric protector. Rohu is a commercially valuable culturable fish species. The present study was undertaken to assess the effects of dietary black seeds on the growth performance and antioxidant status of rohu. Fingerlings were fed on diets containing 0.0%, 1.0% and 2.5% black seed for 28 days. The results showed that rohu fed on black seed supplemented diets has increased growth rate. Moreover, black seed supplementation improved the muscles protein contents and antioxidant status as indicated by decreased lipid peroxidation and increased antioxidant enzymes levels in the liver, kidney, gills and brain of rohu. The black seed fed rohu showed decreased hepatic–nephric marker key-functioning marker enzymes levels. The histo-architecture of liver and kidney remained unchanged following black seed supplementation. Black seed is cheap and locally available in Pakistan. On the basis of the present study results, 2.5% black seed supplementation is suggested in rohu diet to increase its growth and avoid oxidative stress related losses. The results of the present study will be useful for nutritionists, aquaculturists and researchers in formulating aqua feeds.

**Abstract:**

This feeding trial was conducted to investigate the effects of dietary black seed (*Nigella sativa*) supplementation on the growth performance, muscles proximate composition, antioxidant and histo-biochemical parameters of rohu (*Labeo rohita*). Fingerlings (8.503 ± 0.009 g) were fed on 0.0%, 1% and 2.5% black seed supplemented diets for 28 days. Fish sampling was done on the 7th, 14th, 21st and 28th day of experiment. The results of the present study indicated that black seed supplementation significantly increased growth performance and muscles protein contents of rohu over un-supplemented ones. Lipid peroxidation levels significantly decreased in all the studied tissues (liver, gills, kidney and brain) of black seed fed rohu, whereas the antioxidant enzymes (catalase, glutathione-S-transferase, glutathione peroxidase and reduced glutathione) activities were increased in all the studied tissues of black seed supplemented rohu at each sampling day. The hepatic-nephric marker enzymes levels were decreased for black seed fed rohu. The present study showed that tested black seed levels are safe for rohu. Black seed is cheaply available in local markets of Pakistan; therefore, based on the results of the present study, it is suggested that black seed has potential to be used as natural growth promoter and antioxidant in the diet of rohu.

## 1. Introduction

Aquaculture is the fastest-growing animal food producing sector and its products are valuable source of animal protein, omega-3 polyunsaturated fatty acids, vitamins and essential micronutrients. Globally, aquaculture products demand is rapidly increasing, which has led to the intensification of aquaculture practices [1]. Intensification of the aquaculture industry has resulted in a stressful environment, which is responsible for the immunosuppression, oxidative stress and increased risk for infectious diseases in farmed fish [2]. Although several growth promoters, synthetic hormones and chemotherapeutics were being used for enhancing fish yield and for the treatment of diseases [3] (pp. 118–127), their continuous use has posed serious adverse effects on fish, environment and its consumers health [4]. Currently, consumers’ demand for safe and quality farmed fish is increasing, and to meet these demands, researchers have intensified efforts to develop safe fish feed additives/supplements to substitute traditionally used synthetic hormones and chemotherapeutics agents [5].

The supplementation of medicinal plants and their derivatives in aqua feed has attracted a lot of attention globally; therefore, it has become a subject of active scientific investigations [6]. There are more than 60 different medicinal plants species reported to be used in aquaculture industry [7,8,9]. These plants species possess several bioactive constituents and nutrients that make them potent pharmacological and therapeutic agents [10]. Being biodegradable, cheaper, easily available and eco-friendly, medicinal plants are a promising substitute to synthetic hormones and antibiotics that were traditionally used in aquaculture industry [11].

Black seed (*Nigella sativa*) is a medicinal plant belonging to the family ranunculaceae and it has been known as far back as 1400 years ago [12]. Due to its numerous therapeutic properties, black seed is widely cultivated and used in different regions of the world [13,14]. The pharmacological properties of this plant are mainly attributed to its seeds, which have several bioactive constituents such as thymoquinone, dithymoquinone, thymol, nigellicine and nigellidine [15].

Several researchers have reported antifungal [16] (pp. 31–35), antimicrobial [17] (pp. 693–703), antioxidant [18] (pp. 100–106), immunomodulatory [19] (pp. 103–109), hepatoprotective [20] (pp. 1292–1303) and nephroprotective [21] (pp. 692–699) properties of black seed in aquaculture practices. The positive effects of dietary black seed have been shown on the growth performance, biochemical and immuno-haematological parameters of *Carrasius auratus* [22] (pp. 285–290), *Oreochromis niloticus* [23] (pp. 1–4), *Lates calcarifer* [24] (pp. 496–503), *Oncorhynchus mykiss* [25] (pp. 193–197), *Sparus aurata* [26] (pp. 43–50), *Anabas testudineus* [27] (pp. 331–339) and *Cyprinus carpio* [28] (pp. 87–92), but to our best knowledge there is no information available regarding the use of *N. sativa* in rohu (*Labeo rohita*) diet.

Rohu is cultured under semi-intensive polyculture system and is highly prestigious among other culturable carp species; therefore, it is highly demanded as food fish in Pakistan [29]. *N. sativa* is a native medicinal plant species, cultivated in almost all the provinces of Pakistan and, accordingly, it is cheaply available in local markets [30,31,32]. Therefore, the present study was undertaken to investigate the effects of dietary *N. sativa* supplementation on the growth performance, proximate composition, antioxidant and histo-biochemical parameters of rohu fingerlings.

## 2. Materials and Methods

### 2.1. Fish Culture and Diets Preparation

Healthy rohu fingerlings were procured from a government fish seed hatchery in Lahore and acclimatized under laboratory conditions for two weeks prior to this feeding trial. Experimental fish (8.503 ± 0.009 g) were randomly distributed into three groups and kept in well-aerated glass aquaria filled with 50 L of water (60 fingerlings/group). The experiment was done in triplicates and lasted for 28 days. The commercially available floating carps feed prepared using soybean meal and plant protein products, grain and cereal products, yeast, DCP, vitamins and trace minerals, was purchased from Oryza organics, Pvt. Ltd., Lahore, Pakistan, and used as a basal diet for the present study. Black seed was purchased from a local shop in Lahore and grinded into powder form. Three diets containing 0.0%, 1% and 2.5% black seed were prepared by mixing black seed powder into the basal diet and fed to group 1, 2 and 3 fish respectively. The concentrations of black seed used in this study were selected following Bektas et al. [33]. Fish were fed twice a day at the rate of 5% of their wet body weight and ration size was adjusted on weekly basis after each sampling. The three quarters of aquaria water was siphoned daily and replaced by clean well-aerated water.

### 2.2. Water Quality Parameters

During the experimental period, water quality parameters such as dissolved oxygen, temperature and pH were monitored on a daily basis using portable meters (YSI EcoSense DO200A, Yellow Springs Incorporated, Yellow Springs, OH, USA; ADWA, AD1020 pH/mv/ISE). The average water dissolved oxygen, temperature and pH were 5.6 mg L^−1^, 27 °C and 7.8, respectively.

### 2.3. Fish Sampling

Fish sampling was done on a weekly basis (7th, 14th, 21st and 28th day). On each sampling day, 15 fingerlings/group (5 from each replicate) was harvested using a hand net. The fish were anesthetized using clove oil and their length-weight was recorded prior to their dissection. The liver, kidney, gills and brain of experimental fish were excised, cleaned of extraneous tissues in phosphate buffer solution then immediately snap frozen in liquid nitrogen. In the laboratory, all collected tissues were shifted into an ultra-low freezer (−86 °C) until used for further analysis. Fish dorsal muscles were stored at −20 °C until used for their proximate composition analysis.

### 2.4. Preparation of Tissue Homogenates

The tissues homogenates were prepared in 0.1 M sodium phosphate buffer (pH 7.4) using tissue homogenizer (Scilogex, Cromwell Avenue, Rocky Hill, CT, USA). One portion of each tissues homogenate was stored at −20 °C, which was used for assessing lipid peroxidation and remaining portion of homogenates were centrifuged at 4 °C at 13,500 rpm for 30 min (Allegro 64A centrifuge) to obtain post mitochondrial supernatant (PMS). The PMS was stored in clean labeled eppendorf and stored at −20 °C until used for further biochemical assays.

### 2.5. Growth Performance

The data for percentage weight gain (%WG), specific growth rate (SGR), average daily weight gain (ADWG), protein efficiency ratio (PER), condition factor (CF), hepatosomatic index (HSI) and survival rate were calculated [29].

### 2.6. Proximate Composition Analysis

The basal feed and *N. sativa* seeds used in the present study were analyzed for their proximate composition [34]. Fish dorsal muscles were also analyzed for their proximate composition (% moisture, fat, ash and protein contents) [34].

### 2.7. Oxidative Stress and Antioxidant Defense Markers Assessment

The oxidative stress and antioxidant defense enzymes activities were estimated in the liver, kidney, gills and brain of the fish. The lipid peroxidation level was estimated following the protocol of Faheem and Lone [35]. The reaction mixture (1 mL of 10% tissue homogenate, 1 mL of 10% tricholoro acetic acid and 1 mL of 0.67% thiobarbituric acid) was incubated in boiling water bath for 45 min and cooled at room temperature followed by its centrifugation (2500× *g*) at 4 °C for 10 min. The absorbance of supernatant was recorded at 532 nm and values were expressed as nmole of thiobarbituric acid reactive substances (TBARS) formed per gram of tissue. The biochemical analysis for estimating the tissue antioxidant enzymes activities, catalase (CAT), glutathione-S-transferase (GST) and glutathione peroxidase (GPx), was performed using PMS [36].

CAT activity was measured using 1 mL of 10% PMS with equal volumes of 0.1 M sodium phosphate buffer and 0.09 M H_2_O_2_. The decrease in sample absorbance was recorded after every 30 s at 240 nm and CAT activity was expressed as nmol H_2_O_2_ consumed/min/mg of protein. For tissue GST estimation, change in absorbance of reaction mixture (0.1 M sodium phosphate buffer, 1 mM 1-choloro-2,4-dinitrobenzene (CDNB), 1 mM reduced glutathione and 10% PMS) was recorded at 340 nm and values were expressed as nmol of CDNB conjugates formed/min/mg of protein.

The glutathione peroxidase (GPx) activity was estimated by recording the change in absorbance of reaction mixture (0.1 M sodium phosphate buffer, 10 mM EDTA, sodium azide, 1 mM reduced glutathione, 2 mM NADPH, 0.09 M H_2_O_2_, 1 IU/mL glutathione reductase and 10% PMS) at 340 nm and values were expressed as nmoles of NADPH oxidized/minute/mg of protein. The reduced glutathione (GSH) levels were determined by recording absorbance of reaction mixture (0.1 M phosphate buffer (1 mL), 10 Mm DTNB (1 mL) and 1 mL of PMS) at 412 nm and values were expressed as nmol GSH/gram of tissue. The protein contents were estimated using Bradford reagent with bovine serum albumin as standard [36].

### 2.8. Liver and Kidney Functioning Tests

The hepatic-nephric enzymes levels such as alkaline phosphatase (ALP), alanine and aspartate aminotransferase (ALT and AST), urea and creatinine were determined using liver and kidney tissues homogenates respectively. The Randox colorimetric kits (UK) were used for performing all these biochemical analysis and optical density was noted using UV-visible spectrophotometer (Hitachi U-2000) following manufacturer instructions.

### 2.9. Histological Preparations

At the end of experiment, a small portion of liver and kidney was excised and preserved in 10% formaldehyde solution until used for histological preparations [34].

### 2.10. Data Analysis

The obtained data was presented as mean ± S.E.M (standard error of mean). The data was checked for normality and homogeneity of variance by performing Kolmogorov-Smirnov and Levene tests, respectively. Then, data was analyzed by performing ANOVA followed by Tukey’s honest significant difference test to check significant difference between means (*p* < 0.05). All statistical analysis was performed on GraphPad Prism—8.1.0 (325). The data obtained from histological studies was not subjected to any statistical analysis instead visually examined to find any potential difference between black seed supplemented and un-supplemented groups.

### 2.11. Ethical Statement

The study was carried out in accordance with the principles of the Basel Declaration and recommendations in the proceedings of the meeting of Departmental Doctoral Program Committee, Zoology, University of the Punjab, Lahore, Pakistan. The protocol was approved by Punjab University Advanced Studies and Research Board via letter no: D/7566/Acad.

## 3. Results

### 3.1. Growth Performance

The effects of black seed supplemented diets on the growth performance of rohu fingerlings are presented in Table 1. The results indicated that dietary inclusion of different levels of black seed has positive effects on the growth rate of rohu throughout the study period. A significant increase in %WG and SGR was found for rohu fed black seed supplemented diets in comparison with un-supplemented ones at each sampling day. The results also indicated that group-3 rohu (fed with 2.5% black seed supplemented diet) has statistically higher % ADWG and PER when compared with other groups throughout the study period. The CF and HSI index of black seed fed rohu was found in similar with un-supplemented ones. The fish survival rate was 100% for all the studied groups (Table 1).

### 3.2. Proximate Composition of Black Seed, Basal Diet and Dorsal Muscles of Rohu

The proximate composition of *N. sativa* seeds showed 24.7% crude protein, 32.5% crude fat, 4.1% ash and 5.8% moisture contents. The basal diet (0.0% black seed) showed a composition of 12.81% moisture, 20.37% ash, 5.5% fat and 26.88% crude protein levels. The experimental diet (basal diet +1.0% black seed) showed a composition of 12.75% moisture, 20.42% ash, 5.6% fat and 26.89% crude protein levels. The experimental diet (basal diet +2.5% black seed) showed 12.71% moisture, 20.39% ash, 5.5% fat and 26.91% crude protein levels. The results of proximate composition revealed that dietary black seed supplementation has not statistically affected moisture and fat contents in muscles tissue of rohu at each sampling day; however, the ash contents were found to be significantly increased following black seed supplementation at 21st and 28th day of sampling. The crude protein levels in muscles tissue of rohu was found to be significantly increased following dietary black seed supplementation in comparison with control at each sampling point (Table 2).

### 3.3. Antioxidant Status

**Figure 1:**
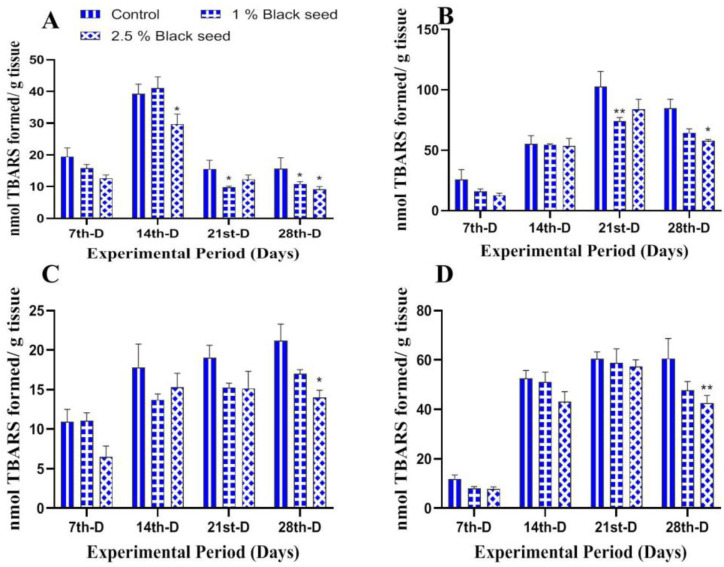
Lipid peroxidation levels in liver (**A**), kidney (**B**), gills (**C**) and brain (**D**) of rohu fed black seed supplemented diets for 28 days. Each value represent the mean ± S.E.M (*n* = 5). Columns with different asterisk are statistically different (* *p* < 0.05 and ** *p* < 0.01).

**Figure 2:**
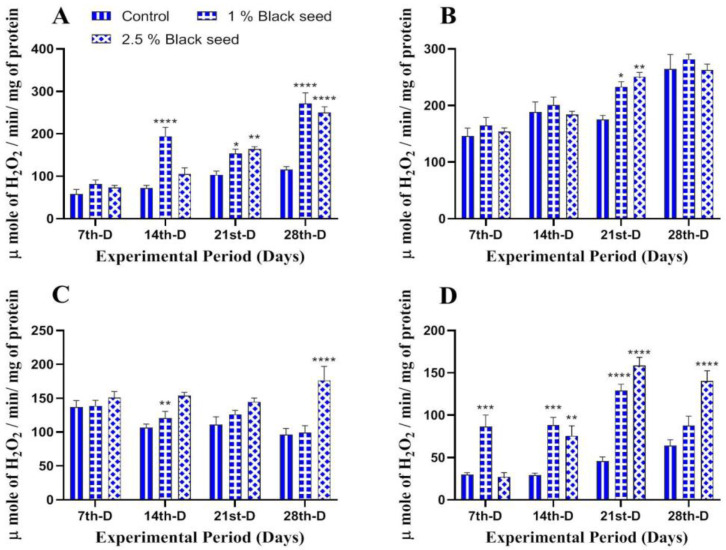
Catalase activity in liver (**A**), kidney (**B**), gills (**C**) and brain (**D**) of rohu fed black seed supplemented diets for 28 days. Each value represent the mean ± S.E.M (*n* = 5). Columns with different asterisk are statistically different (* *p* < 0.05, ** *p* < 0.01, *** *p* < 0.001 and **** *p* < 0.0001).

**Figure 3:**
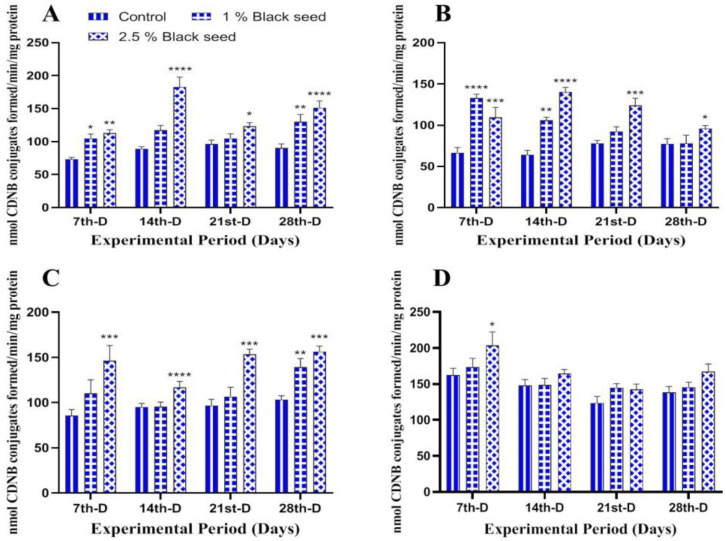
Glutathione-S-transferase activity in liver (**A**), kidney (**B**), gills (**C**) and brain (**D**) of rohu fed black seed supplemented diets for 28 days. Each value represent the mean ± S.E.M (*n* = 5). Columns with different asterisk are statistically different (* *p* < 0.05, ** *p* < 0.01, *** *p* < 0.001 and **** *p* < 0.0001).

**Figure 4:**
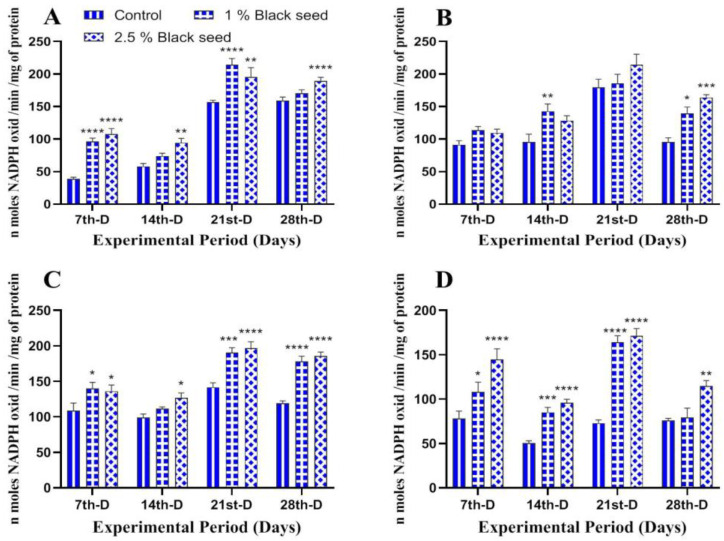
Glutathione peroxidase activity in liver (**A**), kidney (**B**), gills (**C**) and brain (**D**) of rohu fed black seed supplemented diets for 28 days. Each value represent the mean ± S.E.M (*n* = 5). Columns with different asterisk are statistically different (* *p* < 0.05, ** *p* < 0.01, *** *p* < 0.001 and **** *p* < 0.0001).

**Figure 5:**
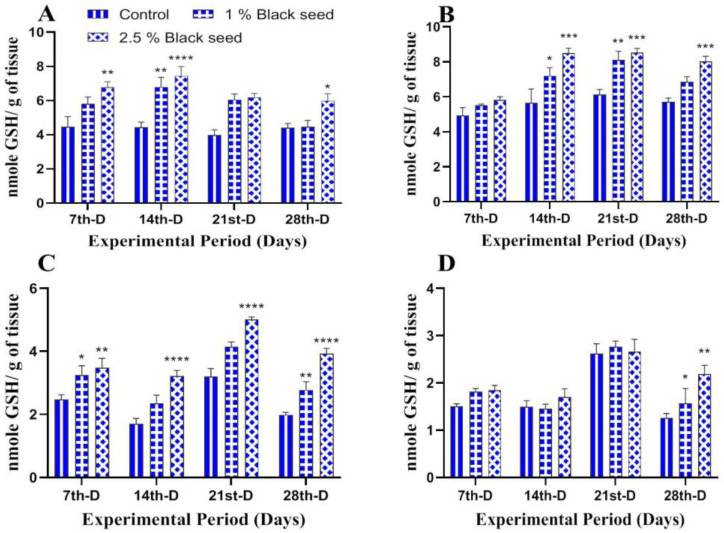
Reduced glutathione levels in liver (**A**), kidney (**B**), gills (**C**) and brain (**D**) of rohu fed black seed supplemented diets for 28 days. Each value represent the mean ± S.E.M (*n* = 5). Columns with different asterisk are statistically different (* *p* < 0.05, ** *p* < 0.01, *** *p* < 0.001 and **** *p* < 0.0001).

The effects of dietary black seed supplementation on the oxidative stress and antioxidant enzymes activities in all the studied tissues of rohu are shown in Figure 1, Figure 2, Figure 3, Figure 4 and Figure 5. The black seed supplemented rohu showed decreased lipid peroxidation levels in all the studied tissues (liver, kidney, gills and brain) when compared with un-supplemented ones (Figure 1A–D).

The antioxidant enzymes (CAT, GST and GPx) was found to be increased in all the studied tissues of rohu fed black seed supplemented diets when compared with rohu fed with basal diet throughout the study period (Figure 2, Figure 3 and Figure 4).

Furthermore, GSH levels were elevated in all the studied tissues of rohu fed with black seed supplemented diets in comparison with control (Figure 5A–D).

### 3.4. Liver and Kidney Histo-Biochemical Parameters

The results indicated decreased hepatic ALP, AST and ALT levels in rohu fed black-seed-supplemented diets in comparison with rohu fed basal diet. Moreover, the dietary black seed supplementation decreased urea and creatinine levels (Table 3). Among the tested concentrations, 2.5% black seed supplementation was found to be most effective.

**Figure 6:**
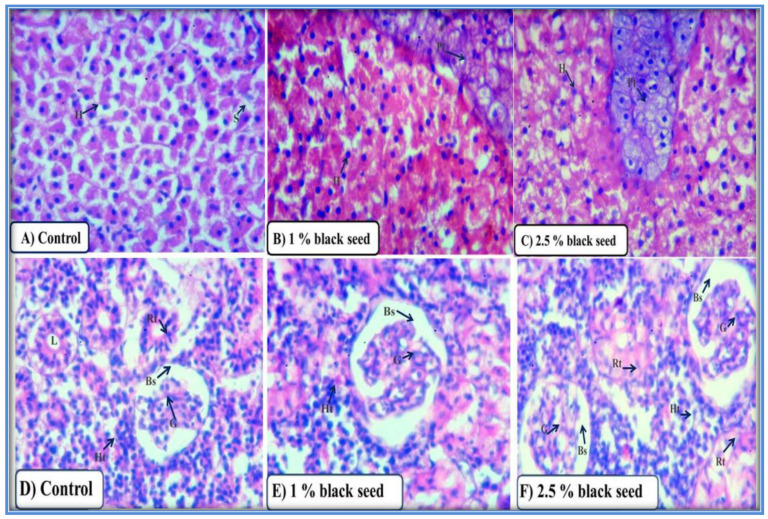
Photomicrographs of liver (**A**–**C**) and kidney (**D**–**F**) sections of rohu from control and black seed supplemented groups at 28th day of the experiment. (H) hepatocytes, (S) sinusoids, (Pt) pancreatic tissue, (G) glomerulus, (Bs) bowmen’s space, (Rt) renal tubule, (L) lumen and (Ht) hematopoietic tissue. H & E, 400X.

The histo-architecture of liver (hepatocytes, sinusoids and pancreatic tissue) and kidney (glomerulus, Bowman’s space, renal tubules and hematopoietic tissue) of rohu fed black seed supplemented diets was in similar with control group (Figure 6A–F).

## 4. Discussion

Medicinal plants have potential to be used as appetizer [37] (pp. 220–227), growth promoter [38] (pp. 217–222), hepatic-nephric protector [39] (504–514), immunomodulator [40] (pp. 346–354) antistress [41] (pp. 19–25) and antioxidants [42] (pp. 277–284) in aquaculture practices. The results of the present study reinforce this view. In the present study, rohu fed with black seed supplemented diets showed higher growth rate when compared with fish fed an un-supplemented diet. Similar to present study results, the growth rate of *Oreochromis niloticus* fed on 2% black seed supplemented diet improved [43]. The growth performance of *Cyprinus carpio* fingerlings fed on 1% black seed supplemented diet enhanced [44]. *Anabas testudineus* fingerlings growth rate was increased when fed on *N. sativa* oil supplemented diets for 30 days [27]. *Onocorhynchus mykiss* juveniles fed on 1% and 1.3% black seed oil supplemented diets (0.1%, 0.4%, 0.7%, 1% and 1.3%) improved their growth rate [45]. The growth rate of *O. mykiss* was significantly increased when fed on 2.5 g Kg^−1^ black seed powder supplemented diet for 25 days [33]. All these aforementioned reports support our results. Black seeds are reported to contain a variety of alkaloids, saponins, minerals (copper, iron, phosphorous and zinc) and vitamins such as folic acid, pyridoxine, riboflavin, vitamin-E and thiamin [46] (239–242); the cumulative effects of all these components might have increased the growth performance of rohu.

The results of the present study showed higher protein efficiency ratio for black seed supplemented rohu at each sampling day. In agreement with present study results, *Oreochromis niloticus* fed black seed meal supplemented diets showed higher protein efficiency ratio [47]. The results of the present study also demonstrated that inclusion of black seed in rohu diets has not altered its hepatosomatic index. Likewise, the hepatosomatic index of *Salmo caspius* fed with 3% garlic supplemented diet remained unchanged [48]. In the present study, muscle protein contents were increased for rohu fed with black seed supplemented diets. The body composition of *Oncorhynchus mykiss* fed on diets supplemented with black seed oil (1 and 1.3%) demonstrated increased protein contents, thus improving its nutritional value [45]. These results are concomitant with our findings. *Dicentrarchus labrax* fed with 1% *Thymus vulgaris* supplemented diet has showed higher protein contents in its fillets [38]. Black seeds are a rich source of several essential amino acids (methionine, threonine, lysine, arginine, glutamic acid, leucine, tyrosine and proline) and (16–19.9%) proteins [49] (pp. 1314–1315). Thus, their inclusion in the diet has improved the muscles protein contents in rohu.

Several environmental stressors are responsible for production of intracellular reactive oxygen species causing oxidative stress in fish, which is indicated by the loss of its antioxidant enzymes activities [36]. There are several types of synthetic antioxidants used as feed additives [50] (pp. 1652–1657); however, accumulation of synthetic antioxidants and their metabolites residues in flesh has increased consumers’ concerns regarding consumption of farmed fish [51]. Therefore, researchers are focusing on plant-based natural antioxidants to replace traditional synthetic antioxidants in fish feed [10]. Medicinal plants are a potential candidate to be used as natural antioxidant in aqua feed. There are significant reports regarding the use of medicinal plants as natural antioxidant in animal nutrition [52] (pp. 89–100) although fewer in aquaculture practices [9].

Lipid peroxidation is an extensively used biomarker to assess oxidative-stress-related damages in animal tissues [35]. In our study, dietary black seed supplementation decreased lipids peroxidation level in all the studied tissues of rohu. *Sparus aurata* fed with *Trigonella foenum graecum* supplemented diets for three weeks showed decreased lipid peroxidation levels in their muscles [42]. Thymoquinone (30–80%) is a major bioactive compound present in black seed which has potential to inhibit iron-dependent lipid peroxidation (Fenton reaction) in a concentration-dependent manner [53]. Enzymatic and non-enzymatic antioxidants such as CAT, GST, GPx and GSH are set to maintain lowest level of reactive oxygen species (ROS) in cells and, therefore, are an essential component of defense response of an organism [54]. The activities of antioxidant enzymes differ among different tissues and were found to be higher in those tissues that have higher oxidative potential [34]. The results of the present study revealed increased CAT, GST, GPx and GSH levels in all the studied tissues of rohu fed black seed supplemented diets. The dietary *Ocimum gratissimum* supplementation increased serum CAT and GST activities in *Clarias gariepinus* [40]. Dietary *Curcuma longa* supplementation for two months increased hepatic GPx and GSH levels in *Ctenopharyngodon idella* [55]. The increased activities of these antioxidant enzymes are attributed to the bioactive phytochemical components found in black seed (O-cymene; 2-isopropylidene-5-methylhex-4-enal; limonen-6-ol, pivalate; longifolene; phenol,4-methoxy-2,3,6-trimethyl; l-(+)-ascorbic acid 2,6-dihexadecanoate and 1-heptatriacotanol), which has free radicals scavenging properties, thus making them a potent antioxidant agent [56].

Liver is an important organ with high metabolic rate. Phosphatases and transaminases are vital for assessing liver function as they regulate several metabolic processes involving synthesis and deamination of amino acid during fluctuating energy demands under different nutritional, physiological and environmental situations [57]. The results of the present study demonstrated decreased hepatic ALP, AST and ALT levels for rohu fed black seed supplemented diets. The dietary *Allium sativum* (2.5% and 5%) supplementation for six weeks has decreased serum ALP and AST levels in *Cyprinus carpio* [58]. The dietary inclusion of *Aloe vera* (0.5%, 1%, 2% and 4%) decreased serum AST and ALT levels in GIFT tilapia [39]. These reports are in agreement with our results. The hepatoprotective action of black seed has been largely attributed to its thymoquinone contents, which protects the liver from injuries through different mechanisms such as increased activity of antioxidant enzymes, inhibition of iron dependent lipid peroxidation and lipogenesis in the hepatocytes [59].

Nitrogenous products such as urea, uric acid and creatinine are useful indicators for evaluating the state of the kidney and gills of fish. Among all of these, creatinine is most important as it represents more than 50% of nitrogenous waste excreted through the fish kidney [60]. In the present study, nephroprotective effects of black seed have been elucidated by decreased creatinine and urea levels in rohu fed black seed supplemented diets over un-supplemented ones. The inclusion of black seed in the diet of *Lates calcarifer* has decreased its serum creatinine level [24]. The herbal (*Thymus vulgaris* and *Rosmarinus officinalis*) supplemented diets has decreased serum urea and creatinine levels in *Dicentrarchus labrax* juveniles for 45 days [38]. The hepatic-nephric beneficial effects of black seed were further confirmed by regular liver and kidney histoarchitecture of rohu fed supplemented diets in similar with un-supplemented ones. The bioactive ingredients (carvacrol, α-tocopherol, thymoquinone and nigellicine) abundantly found in black seed are ascribed to scavenge ROS, thereby maintaining normal histoarchitecture and metabolic enzymes levels [61].

## 5. Conclusions

In conclusion, the results of the present study gave a new insight on the use of black seed as natural growth promoter, antioxidant and hepatic-nephric protector in aqua feed of rohu. The results of the present study elucidated that all the studied levels of black seed (1% and 2.5%) are safe and have positive effects on the growth performance, antioxidant and histo-biochemical parameters of rohu. Black seed is suggested as a potential candidate to be used as feed additive in intensive fish culturing practices to avoid stress-related losses and ultimately to enhance the overall fish production.

## Figures and Tables

**Table 1:** Growth performance of rohu fingerlings fed varying levels of black seed supplemented diets for 28 days. Values are represented as mean ± S.E.M (*n* = 15).

Parameters	Sampling Period (Days)	Dietary Black Seed Levels		
		Group-I (0%)	Group-II (1%)	Group-III (2.5%)
**Initial weight (g/fish)**	0-D	8.486 ± 0.138	8.517 ± 0.355	8.506 ± 0.228
**Final weight** **(g/fish)**	7th-D	8.825 ± 0.136	9.693 ± 0.390	9.784 ± 0.246 *
	14th-D	9.540 ± 0.178	11.32 ± 0.387 ***	11.44 ± 0.297 ***
	21st-D	10.11 ± 0.244	11.29 ± 0.183 **	12.13 ± 0.204 ***
	28th-D	11.27 ± 0.180	11.84 ± 0.282 **	12.55 ± 0.206 ***
**%WG**	7th-D	4.056 ± 0.154	14.13 ± 1.514 ****	15.44 ± 1.927 ****
	14th-D	12.58 ± 1.511	22.65 ± 1.700 ****	34.77 ± 1.618 ****
	21st-D	22.96 ± 1.628	34.00 ± 2.012 ****	36.08 ± 1.626 ****
	28th-D	32.03 ± 1.648	37.96 ± 2.043 ****	40.50 ± 1.660 **
**SGR (%/day)**	7th-D	0.567 ± 0.021	1.866 ± 0.183 ***	2.015 ± 0.231 ***
	14th-D	1.667 ± 0.201	4.152 ± 0.208 ****	4.243 ± 0.170 ****
	21st-D	2.927 ± 0.197	2.892 ± 0.191 *	4.382 ± 0.169 ****
	28th-D	4.055 ± 0.182	4.253 ± 0.209 *	4.839 ± 0.168 *
**ADWG**	7th-D	0.048 ± 0.001	0.168 ± 0.016 ****	0.182 ± 0.021 ****
	14th-D	0.151 ± 0.017	0.401 ± 0.017 ****	0.419 ± 0.021 ****
	21st-D	0.276 ± 0.018	0.412 ± 0.016	0.435 ± 0.021 ****
	28t-D	0.397 ± 0.017	0.437 ± 0.016	0.488 ± 0.021 ****
**PER**	7th-D	0.012 ± 0.001	0.034 ± 0.004	0.047 ± 0.005 ****
	14th-D	0.039 ± 0.004	0.104 ± 0.003	0.109 ± 0.005 ****
	21st-D	0.072 ± 0.005	0.069 ± 0.004	0.113 ± 0.005 ****
	28th-D	0.103 ± 0.005	0.107 ± 0.004	0.127 ± 0.005 **
**CF (g/cm^3^)**	7th-D	1.151 ± 0.059	1.176 ± 0.087	1.004 ± 0.049
	14th-D	1.411 ± 0.087	1.568 ± 0.150	1.283 ± 0.097
	21st-D	1.507 ± 0.092	1.426 ± 0.110	1.327 ± 0.107
	28th-D	1.538 ± 0.071	1.585 ± 0.137	1.360 ± 0.107
**HSI (%)**	7th-D	1.529 ± 0.076	1.536 ± 0.089	1.543 ± 0.075
	14th-D	1.534 ± 0.069	1.537 ± 0.112	1.545 ± 0.067
	21st-D	1.549 ± 0.081	1.557 ± 0.064	1.562 ± 0.0452
	28th-D	1.558 ± 0.054	1.568 ± 0.086	1.575 ± 0.060
**Survival rate (%)**	7th–28th-D	100	100	100

Values with the * in the same row are statistically different (* *p* < 0.05, ** *p* < 0.01, *** *p* < 0.001 and **** *p* < 0.0001). where, Percentage Weight Gain, **%WG** = 100 × (final body weight—initial body weight)/Initial body weight; Specific Growth Rate, **SGR** = 100 × ln (final body weight/Initial body weight)/days of the experiment; Average Daily Weight Gain, **ADWG** = (final body weight—initial body weight)/days of the experiment; Protein Efficiency Ratio, **PER** = weight gain/protein intake; Condition Factor, **CF** = 100 × body weight (g)/body length (cm^3^); Hepatosomatic Index, **HSI** = 100 × liver weight/body weight and Percentage Survival Rate, **% SR** = 100 × final number of fish/initial number of fish.

**Table 2:** Effect of dietary black seed supplementation on the dorsal muscles proximate composition of rohu for 28 days. Values are represented as mean ± S.E.M (*n* = 5).

Parameters	Sampling Period (Days)	Dietary Black Seed Levels		
		Group-I (0%)	Group-II (1%)	Group-III (2.5%)
**Moisture** **(%)**	7th-D	74.61 ± 0.105	74.46 ± 0.065	73.99 ± 0.025
	14th-D	74.31 ± 0.105	74.35 ± 0.070	73.92 ± 0.045
	21st-D	74.25 ± 0.255	74.28 ± 0.050	73.78 ± 0.115
	28th-D	74.08 ± 0.095	74.07 ± 0.050	73.73 ± 0.160
**Fat** **(%)**	7th-D	3.100 ± 0.100	3.035 ± 0.065	2.915 ± 0.025
	14th-D	3.050 ± 0.050	3.010 ± 0.030	2.980 ± 0.010
	21st-D	2.995 ± 0.015	2.915 ± 0.045	2.910 ± 0.050
	28th-D	3.025 ± 0.075	2.900 ± 0.040	2.825 ± 0.065
**Ash** **(%)**	7th-D	2.655 ± 0.005	2.925 ± 0.055	2.855 ± 0.025
	14th-D	2.855 ± 0.015	2.950 ± 0.030	2.970 ± 0.010
	21st-D	2.815 ± 0.055	2.925 ± 0.035 *	2.945 ± 0.035 *
	28th-D	2.845 ± 0.025	2.930 ± 0.010 **	2.990 ± 0.030 **
**Protein** **(%)**	7th-D	16.48 ± 0.050	16.79 ± 0.005 *	16.90 ± 0.015 **
	14th-D	16.64 ± 0.025	16.93 ± 0.045 **	17.00 ± 0.010 **
	21st-D	16.67 ± 0.075	16.90 ± 0.080 *	17.08 ± 0.045 *
	28th-D	16.76 ± 0.080	17.00 ± 0.010 *	17.28 ± 0.005 **

Means with the * in the same row are statistically different (* *p* < 0.05 and ** *p* < 0.01).

**Table 3:** Effects of dietary black seed supplementation on the hepatic-nephric key-functioning enzymes levels in rohu for 28 days. Values are represented as mean ± S.E.M (*n* = 5).

Parameters	Sampling Period (Days)	Dietary Black Seed Levels		
		Group-I (0%)	Group-II (1%)	Group-III (2.5%)
**ALP** **(U/l)**	7th-D	460.0 ± 0.512	322.0 ± 0.018 **	384.9 ± 0.047
	14th-D	500.5 ± 0.123	350.2 ± 0.312 **	390.4 ± 0.051 *
	21st-D	510.5 ± 0.145	440.1 ± 0.541 *	386.7 ± 0.110 *
	28th-D	548.9 ± 0.312	413.7 ± 0.113 **	417.7 ± 0.111 **
**ALT** **(U/l)**	7th-D	24.81 ± 0.013	24.36 ± 0.045	27.72 ± 0.110
	14th-D	25.61 ± 0.009	23.64 ± 0.020	28.75 ± 0.009
	21st-D	26.01 ± 0.101	22.08 ± 0.011	21.11 ± 0.017
	28th-D	26.38 ± 0.002	26.01 ± 0.013	25.22 ± 0.005
**AST** **(U/l)**	7th-D	26.77 ± 1.012	26.48 ± 0.016	22.98 ± 0.079
	14th-D	27.35 ± 1.000	22.57 ± 0.078	18.07 ± 0.065
	21st-D	27.94 ± 0.998	27.40 ± 0.015	25.15 ± 0.103 *
	28th-D	28.52 ± 1.145	22.65 ± 0.046	21.69 ± 0.078 *
**Urea** **(mg/dl)**	7th-D	2.963 ± 0.213	2.618 ± 0.167	2.550 ± 0.078
	14th-D	3.376 ± 0.136	2.481 ± 0.210	2.687 ± 0.106
	21st-D	3.032 ± 0.178	2.825 ± 0.198	2.550 ± 0.117
	28th-D	3.032 ± 0.145	2.550 ± 0.166	2.274 ± 0.178 *
**Creatinine** **(mg/dl)**	7th-D	0.152 ± 0.056	0.143 ± 0.045	0.118 ± 0.012
	14th-D	0.168 ± 0.033	0.134 ± 0.034	0.125 ± 0.036 *
	21st-D	0.172 ± 0.098	0.125 ± 0.076	0.110 ± 0.067 **
	28th-D	0.152 ± 0.097	0.116 ± 0.045 *	0.089 ± 0.045 **

Means with the * in the same row are statistically different (* *p* < 0.05 and ** *p* < 0.01).

## Data Availability

The data presented in this study is available within the article.
